# Supplementary material for: A molecular mechanism for the topographic alignment of convergent neural maps
Source: eLife. 2017 Mar 14;6:e20470. doi: 10.7554/eLife.20470 (PMC5360444; doi:10.7554/eLife.20470)
Supplement: Source code 1. — DOI: http://dx.doi.org/10.7554/eLife.20470.020 [file elife-20470-code1.pdf]

## Leave-One-Out Method Single Map

```
## 2014-05-28
dat <- read.csv('FileName')

x <- dat[,1]
y <- dat[,2]
tiff(file='FileName',width = 4.5, height = 5, units = 'in', res = 600)
plot(x, y, xlim=c(0,100), ylim=c(0,100), pch=20,cex=1, bty='n',
    xlab='V1 axis', ylab='collicular axis', main='CC map',las=1)
## default for f is 2/3
##f <- 1/10
f <- 2/3
l <- lowess(x, y, f=f)
lines(l)

## LOO
npts <- length(x)
for (i in 1:npts) { l2 <- lowess(x[-i], y[-i], f=f)  lines(l2, col='grey')}
lines(l, lwd='2')
dev.off()
```

## Leave-One-Out Method Double Map

```
## 2014-05-28
dat <- read.csv('FileName')
x <- dat[,1]
y <- dat[,2]
y1 <- dat[,3]
```

```
tiff(file=’FileName’,width = 4.5, height = 5, units = 'in', res = 600)
plot(x, y, xlim=c(0,100), ylim=c(0,100), pch=20,cex=1, bty='n', col='red',
   xlab='V1 axis', ylab='collicular axis', main='CC map',las=1)
points (x, y1, pch=20, cex=1, col='red' )

## default for f is 2/3
##f <- 1/10
f <- 2/3
l <- lowess(x, y, f=f)
l1 <- lowess(x, y1, f=f)
lines(l)

## LOO
npts <- length(x)
for (i in 1:npts) { l2 <- lowess(x[-i], y[-i], f=f)  lines(l2, col='grey')}
lines(l, lwd='2', col='red')

##LOO 2
npts <- length(x)
for (i in 1:npts) { l2 <- lowess(x[-i], y1[-i], f=f)  lines(l2, col='grey')}
lines(l1, lwd='2', col='red')

dev.off()
```
