# Supplementary material for: A molecular mechanism for the topographic alignment of convergent neural maps
Source: eLife. 2017 Mar 14;6:e20470. doi: 10.7554/eLife.20470 (PMC5360444; doi:10.7554/eLife.20470)
Supplement: Source code 2. — DOI: http://dx.doi.org/10.7554/eLife.20470.021 [file elife-20470-code2.docx]

**3-Step Map Alignment Model: Generates this Retino-collicular and Cortico-collicular mapping sequentially based on Correlated Neuronal Activity and EphA/ephrin-As gradients**

Copyright (C) 2017 Michael Reber (michael.reber@inserm.fr)

This program is free software: you can redistribute it and/or modify it under the terms of the GNU general Public License as published by the Free Software Foundation, either version 3 of the License, or (at your option) any later version.

This program is distributed in the hope that it will be useful, but WITHOUT ANY WARRANTY; without even the implied warranty of MERCHANTABILITY or FITNESS FOR A PARTICULAR PURPOSE. See the GNU General Public License for more details.

You should have received a copy of the GNU General Public License along with this program. If not, see <http://www.gnu.org/licenses/>.

https://github.com/michaelreber/3-step-Map-Aligment-Model

**3-step alignment model Matlab code**

'Genotype can be: Wildtype, Heterozygote or Homozygote'

n = input('Genotype: ');

switch n

case 'Wildtype'

ephrinA3KI=0

Genotype='Wildtype'

threshold=5.68

case 'Heterozygote'

ephrinA3KI=0.22

threshold=5.68

Genotype='Heterozygote'

case 'Homozygote'

ephrinA3KI=0.44

threshold=8.68

Genotype='Homozygote'

otherwise

disp('Invalid genotype')

end

% 1D simulation of the development of retinocollicular maps in wild-type species

% From Koulakov and Tsigankov 2004

%Intial parameters

NIterations =10000000 %number of measurement of the axon distribution

N = 100; %number of axons

alpha = 200; %simulation parameter, the transition probability coefficient in rostral-caudal direction

gamma = 1; % the overall strength of the activity-dependent component

%Constants for the activity-dependent portion

d=3;%Interaction distance in the superior colliculus

R=0.11*N;%Interaction distance in the retina

figure

%Generation of the retinocollicular map

%settting receptor concentration profiles

%Retinal EphA

X=linspace(0,100,100);

RA = (0.26*exp(0.023*X))+1.05; %Equation from Reber et al., 2004

RA(RA<0)=0;

%Plotting retinal EphA gradient

subplot(2,4,1)

a=area(RA);

a.FaceColor=[.5 .5 .5];

a.EdgeColor=[.5 .5 .5];

set(gca,'XTick',[0,20,40,60,80,100]);

set(gca,'XtickLabel',{'Nasal';'20'; '40'; '60';'80';'Temporal'});

xlabel('Retina position (%)');

ylabel('EphA concentration');

title('EphA level in the retina');

%Collicular ephrinA

X=linspace(0,100,100);

LA = exp((X-N)/N)-exp((-X-N)/N); %Equation from Tsigankov and Koulakov, 2010

LA(LA<0)=0;

%Plotting collicular ephrinA gradient

subplot(2,4,2)

b=area(LA);

b.FaceColor=[.8 .8 .8];

b.EdgeColor=[.8 .8 .8];

set(gca,'XTick',[0,20,40,60,80,100])

set(gca,'XtickLabel',{'Rostral';'20'; '40'; '60';'80';'Caudal'})

axis([0 100 0 1])

xlabel('SC position (%)')

ylabel('Relative ephrinA concentration')

title('EphrinA level in the SC')

%setting initial configuration

RofL = randperm(N);

LofR = zeros(N,1);

for i=1:N

LofR(i) = find(RofL == i);

end

%simulation of development process for the retinocollicular map

for t = 1:NIterations%(iterator)

%Specify the coordinates of the axons in the SC.

iL = ceil(rand*(N)); % ceil rounds up towards an integer.

iL2 = ceil(rand*(N)); % Picks second axon NT index.

% Calculate Echem

EA=alpha* ( RA(RofL(iL2)) - RA(RofL(iL)) ) * (LA(iL2)-LA(iL));

Echem=EA;

% Source position

RetiL=RofL(iL);

RetiL2=RofL(iL2);

%Calculate C with RetiL and RetiL2

Rret=abs(RetiL-RetiL2);%

C=exp(-Rret/R);

%Calculate U with iL and iL2

Rsc_sq=(iL-iL2)^2;

U=exp(-Rsc_sq/(2*d^2));

EactA= U.*C;

%This is the change in Eact.

Eact=-(gamma/2)*(sum(sum(EactA)));

% Calculate Etot

Etot= Eact+Echem;

p=1/(1+exp(4*Etot));

if (rand < p) %Switches the two axons

tmp = LofR(RofL(iL)); %Extracting the position of the first axon

LofR(RofL(iL)) = LofR(RofL(iL2)); %Replacing it with the position of the second axon

LofR(RofL(iL2)) = tmp; %Putting the first axon into the former

%position of the second axon

tmp = RofL(iL); % Extracts the value of the first axon

RofL(iL) = RofL(iL2); %Replacing it with the value of the

% second axon

RofL(iL2) = tmp; %Putting the first axon's value into the second

%axon

end

end

MapLR = zeros(N,N);

for j=1:N

MapLR(j,N+1-RofL(N+1-j)) = 1;

end

%Extracting indexes

inds = find(MapLR==1);

[row, col] = ind2sub(size(MapLR),inds);

%Plotting the retinocollicular map

subplot(2,4,3)

scatter(col,row,'filled');

s=scatter(col,row,'filled');

s.MarkerFaceColor = [0.5 0.5 0.5];

set(gca,'XDir','Reverse');

set(gca,'XTick',[0,20,40,60,80,100]);

set(gca,'XtickLabel',{'Temporal';'80'; '60'; '40';'20';'Nasal'})

set(gca,'YTick',[0,20,40,60,80,100]);

set(gca,'YtickLabel',{'Rostral';'20'; '40'; '60';'80';'Caudal'})

xlabel('Retina position (%)')

ylabel('SC position (%)')

title([Genotype ' Retinocollicular map']);

%Generation of the corticocollicular map

%Generating the cortical EphA gradient

X = 1:N;

RASC =(exp(-X/N)-exp((X-2*N)/N))+1; %Equation from Tsigankov and Koulakov, 2010 plus constant

RASC=fliplr(RASC)

RASC(RASC<0)=0;

%Plotting the cortical EphA gradient

subplot(2,4,5)

a=area(RASC);

a.FaceColor=[.5 .5 .5];

a.EdgeColor=[.5 .5 .5];

set(gca,'XTick',[0,20,40,60,80,100]);

set(gca,'XtickLabel',{'Medial';'20'; '40'; '60';'80';'Lateral'});

xlabel('Cortical position (%)');

ylabel('Relative EphA concentration');

title('EphA level in V1');

%Generating the retinal ephrinA gradient: Exponential fit of ISH data (unpublished data, Reber)

X=linspace(0,100,100);

gA2=0.5462*exp(0.008403*X); %ephrinA2

gA3=0.44;%ephrinA3

gA5=0.5612*exp(0.01447*X);%ephrinA5

gA5=gA5/1.2 % 20% used for axon-axon interaction

gEphrins=gA2+gA5+gA3; %summed ephrinAs

ISL2=[1,0]

ISL2=repmat(ISL2,1,50)

N3=ISL2.*ephrinA3KI

gEphrins=plus(gEphrins,N3)

%Transposing retinal ephrinAs to the SC

[RCaxis,col2] = find(MapLR>0); %Extracting indexes

LASC(RCaxis)=gEphrins; %Attributing ephrinA values to the positions in the retinocollicular map

%Plotting the collicular ephrinA gradient

subplot(2,4,6)

b=area(LASC);

b.FaceColor=[.8 .8 .8];

b.EdgeColor=[.8 .8 .8];

set(gca,'XTick',[0,20,40,60,80,100])

set(gca,'XtickLabel',{'Rostral';'20'; '40'; '60';'80';'Caudal'})

axis([0 100 0 4.5])

xlabel('Collicular position (%)')

ylabel('Relative ephrinA concentration')

title('Transposed ephrinA level in the SC')

%setting initial configuration

RofL2 = randperm(N);

LofR2 = zeros(N,1);

for i=1:N

LofR2(i) = find(RofL2 == i);

end

%simulation of development process for the retinocollicular map

for t = 1:NIterations%(iterator)

%Specify the coordinates of the axons in the SC.

iL = ceil(rand*(N)); % ceil rounds up towards an integer.

iL2 = ceil(rand*(N)); % Picks second axon NT index.

% Calculate Echem

EA=alpha* ( RASC(RofL2(iL2)) - RASC(RofL2(iL)) ) * (LASC(iL2)-LASC(iL));

Echem=EA;

% Source position

RetiL=RofL2(iL);

RetiL2=RofL2(iL2);

%Calculate C with RetiL and RetiL2

Rret=abs(RetiL-RetiL2);%

C=exp(-Rret/R);

%Calculate U with iL and iL2

Rsc_sq=(iL-iL2)^2;

U=exp(-Rsc_sq/(2*(d^2)));

EactA= U.*C;

%This is the change in Eact.

Eact=-(gamma/2)*(sum(sum(EactA)));

% Calculate Etot

Etot= Eact+Echem;

p=1/(1+exp(4*Etot));

if (rand < p) %Switches the two axons

tmp = LofR2(RofL2(iL)); %Extracting the position of the first axon

LofR2(RofL2(iL)) = LofR2(RofL2(iL2)); %Replacing it with the position of the second axon

LofR2(RofL2(iL2)) = tmp; %Putting the first axon into the former

%position of the second axon

tmp = RofL2(iL); % Extracts the value of the first axon

RofL2(iL) = RofL2(iL2); %Replacing it with the value of the

% second axon

RofL2(iL2) = tmp; %Putting the first axon's value into the second

%axon

end

end

MapLRSC = zeros(N,N);

for j=1:N

MapLRSC(j,N+1-RofL2(N+1-j)) = 1;

end

%Extracting indexes

inds = find(MapLRSC==1);

[row2, col2] = ind2sub(size(MapLRSC),inds);

%Plotting the theoretical map

subplot(2,4,7)

s=scatter(col2,row2,'filled');

s.MarkerFaceColor = [0.5 0.5 0.5];

set(gca,'XTick',[0,20,40,60,80,100]);

set(gca,'XDir','Reverse');

set(gca,'YtickLabel',{'Caudal';'80'; '60'; '40';'20';'Rostral'})

set(gca,'YTick',[0,20,40,60,80,100]);

set(gca,'YDir','Reverse');

set(gca,'XtickLabel',{'Lateral';'80'; '60'; '40';'20';'Medial'})

title([Genotype ' Corticocollicular map']);

%Calculate linear regression

p = polyfit(col2,row2,1);

yfit = polyval(p,col2);

hold on;

%Plot linear regression and upper and lower bound

plot(yfit,'k');

axis([0 100 0 100]);

%Calculating residuals

resid = row2 - yfit;

[Z,mean,theostdev] = zscore(resid);

absresid=abs(resid);

duplication = find(absresid>threshold);

DuplicationPercentage=length(duplication)

%Plotting upper and lower bound

uplim=yfit+threshold;

lowlim=yfit-threshold;

plot(uplim,'k');

plot(lowlim,'k');

set(gca,'XDir','Reverse');

set(gca,'XtickLabel',{'Medial';'80'; '60'; '40';'20';'Lateral'});

%Plotting the residuals

subplot(2,4,8);

axis([0 100 -20 20])

z=ones(1,100);

s1=scatter(col2,resid,'filled');

s1.MarkerFaceColor = [0.5 0.5 0.5];

hold on;

mean=mean.*z;

plot(mean,'k')

threshold=threshold.*z;

plot(threshold,'k')

plot(-threshold,'k')

xlabel('Cortical position (%)');

ylabel('Residuals');

title(['Residuals and duplication percentage:' num2str(DuplicationPercentage)])
