# Supplementary material for: A molecular mechanism for the topographic alignment of convergent neural maps
Source: eLife. 2017 Mar 14;6:e20470. doi: 10.7554/eLife.20470 (PMC5360444; doi:10.7554/eLife.20470)
Supplement: Supplementary file 1. — DOI: http://dx.doi.org/10.7554/eLife.20470.022 [file elife-20470-supp1.docx]

Supplementary Files R2

Primers sequences for genotyping are:

*Efna3* forward: GGTCTTTGAGCAGGTAATCCAGGAG

*Efna3* reverse 1: CCGGAGATTTTTCAGAGCTGGAATGG (WT allele)

*Efna3* reverse 2: GCACGAGCAGCAGCAGCAGAAG (mutant allele)

*Epha4* forward 1: TCC TCG TGC TTT ACG GTA TC (mutant allele)

*Epha4* forward 2: ACC GTT CGA AAT CTA GCC CAG T (WT allele)

*Epha4* reverse: AGC CTT GCC ACC TGG AGC

*Epha3* forward: CTC CAG GGA CTA ACT GGG AC

*Epha3* reverse 1: ACA AGG GCC GGA GAT TTT TCA GAG (WT allele)

*Epha3* reverse 2: GCT CCC GAT TCG CAG CGC ATC G (mutant allele)

Primer sequences for Q-PCR are:

*Efna2* (forward: TCCCCCTTGATCATGTGACCT, reverse: GGTAGGTAGCTCCCCTTCCT), *Efna5* (forward: TTGATGGGTACAGTGCCTGC, reverse: TTCCGAGAACTTCAGCGGTC), *Efna3* (forward: TATGAATTCCATGCCGGCCAA, reverse: CAGACGAACACCTTCATCCT),

*Epha4* (forward: GAGGCTCCTGTGTCAACAACT, reverse: AGTTGCCAATGGGTACCAGC), *Epha7* (forward: TCCTCCTTAGTCGAGGTCCG, reverse: GCCACTCTCCTTCTGCACTG)

*Gapdh* (forward: ACCACAGTCCATGCCATCAC, reverse: TCCACCACCCTGTTGCTGTA),

*Hprt* (forward: CACAGGACTAGAACACCTGC, reverse: GCTGGTGAAAAGGACCTCT).
